# Severe Adverse Drug Reaction to Gadobenate Dimeglumine

**DOI:** 10.1100/tsw.2009.41

**Published:** 2009-05-22

**Authors:** Benjamin D. Singer, Robert S. Woodrick, Jennifer B. Pedicano

**Affiliations:** ^1^ Department of Medicine, Northwestern Memorial Hospital, 251 E. Huron St., Galter Suite 3-150, Chicago, IL 60611, USA; ^2^ Chicago, IL, USA

**Keywords:** gadolinium, gadobenate dimeglumine, anaphylaxis, anaphylactoid reaction

## Abstract

A 57-year-old man was admitted to our hospital for evaluation and management of stroke in the setting of an atrial septal defect. Shortly after receiving gadobenate dimeglumine for magnetic resonance imaging of the pelvic vessels, he experienced cardiac arrest from which he was resuscitated. His course was complicated by profound distributive shock. The presumed cause was a severe anaphylactic or anaphylactoid reaction to the gadolinium-based compound that he received.

## CASE STUDY

Anaphylactoid reactions to iodinated radiocontrast media have been well described[1]. However, anaphylactoid or anaphylactic reactions to gadolinium-based contrast media are exceedingly rare. The reported risk of allergic-like reactions is on the order of 0.01%; fewer than 10% of such reactions rank as severe[2,3,4,5,6].

Gadolinium is a paramagnetic rare-earth metal that enhances the contrast of images obtained by magnetic resonance imaging (MRI). Gadolinium ion ($Gd^{3+}$) is highly toxic; therefore, gadolinium is administered as an aqueous solution of organic chelates. Following intravenous injection, these compounds rapidly distribute to the extracellular space. Gadolinium chelates may be considered drugs, although they are not used for the purpose of altering human biochemistry or physiology. Rather, their paramagnetic properties are employed to increase the accuracy of MRI.

Gadobenate dimeglumine is an organic octadentate gadolinium chelate, which is marketed as MultiHance® by Bracco Diagnostics Inc. MultiHance® contains only gadobenate dimeglumine and water[5]. It is excreted unchanged via the kidneys with an elimination half-life of approximately 1 h in patients with normal renal function[5,7]. The substance has minimal hepatobiliary excretion, but does enhance images of the liver and bile duct system. Use of gadolinium chelates, including gadobenate dimeglumine, is widespread and appears to be increasing with worldwide availability of MRI.

While a single case report exists of an anaphylactic reaction to gadobenate dimeglumine[8], anaphylactic and anaphylactoid reactions have been described in association with other gadolinium chelates[9,10,11,12,13]. These reports suggest cross-reactivity and that a class effect may be present. Although free gadolinium ion has not been recovered in the blood of subjects receiving an intravenous injection of gadobenate dimeglumine[5], it is unclear whether reactions occur to $Gd^{3+}$ or its organic chelator.

Here we present a 57-year-old man who experienced cardiac arrest and profound distributive shock following administration of gadobenate dimeglumine.

The patient was admitted to an outside hospital after a paradoxical embolic stroke via a secundum-type atrial septal defect. His past medical history included gout, depression, benign prostatic hyperplasia, and allergic rhinitis. He had no history of kidney or liver disease. His home medications were allopurinol, sertraline, terazosin, and fluticasone nasal spray. He reported a remote history of lip swelling following administration of iodinated radiocontrast media.

On admission to the outside hospital, he received aspirin daily for stroke and a continuous unfractionated heparin infusion for suspected pelvic deep venous thrombosis. His home medications were continued. Gadopentetate dimeglumine was administered for MRI of the brain; he had no adverse reaction to this agent. The patient was transferred to our hospital 1 week later for further evaluation and management.

Within 2 min following injection of gadobenate dimeglumine for magnetic resonance venography of the pelvis, the patient complained of shortness of breath and feeling warm. His skin became diffusely erythematous with urticarial lesions. He was found to be hypoxemic and then pulseless.

Advanced cardiac life support was initiated. He underwent direct endotracheal intubation; difficulty compressing the ventilation bag was noted. Circulatory evaluation revealed cardiac arrest with pulseless electrical activity. He received intravenous boluses of epinephrine, atropine, and methylprednisolone. After 48 min, the patient regained a pulse and was transferred to the intensive care unit, receiving infusions of epinephrine and dopamine to maintain his mean arterial blood pressure above 50 mmHg. Postresuscitation transesophageal echocardiography demonstrated normal left and right ventricular size, wall motion, and systolic function without evidence of pericardial effusion.

Volume expansion with normal saline and the addition of norepinephrine, vasopressin, and phenylephrine by infusion were required for persistent hypotension. He received intravenous diphenhydramine, famotidine, and methylprednisolone as well as albuterol-ipratropium nebulized solution.

Vasopressor, steroid, antihistamine, and bronchodilator support were weaned off over the following 4 days. His mental status gradually improved from coma to alert, oriented, and conversant by the time of discharge on hospital day 35.

We believe cardiac arrest associated with profound distributive shock resulted from an immediate hypersensitivity reaction (anaphylaxis) or an anaphylactoid reaction to gadobenate dimeglumine. Gadopentetate dimeglumine, which he received 1 week earlier, may have served as a sensitizing substance. An increased likelihood of hypersensitivity to gadolinium-based contrast media has been described in patients with a history of hypersensitivity to iodinated radiocontrast media[3]. Our patient may have been an example of this phenomenon. The precise pathophysiologic mechanism underlying these events could be clarified by skin prick and intradermal testing; however, the patient declined.

While case reports exist of anaphylactic or anaphylactoid reactions to other gadolinium-based contrast media, we believe this is the second case report in the English literature of such a reaction following administration of gadobenate dimeglumine. Although this dramatic presentation ranks on the severe end of the adverse drug reaction spectrum, it highlights the need to remain vigilant when using common, usually benign, agents such as gadolinium chelates.
